# Associations of the Big Five and locus of control with problem gambling in a large Australian sample

**DOI:** 10.1371/journal.pone.0253046

**Published:** 2021-06-14

**Authors:** Juliane M. von der Heiden, Boris Egloff

**Affiliations:** Department of Psychology, Johannes Gutenberg University Mainz, Mainz, Germany; Aalborg University, DENMARK

## Abstract

Gambling may range from being a recreational leisure activity to a behavioral addiction. A rising number of gamblers experience adverse consequences from gambling, termed problem gambling, which may become a challenge for the individual and society. With the present research, we aimed to investigate the correlates of problem gambling. We used a large sample of more than 12,500 individuals (46% male, *M*_age_ = 48, *SD*_*age*_ = 18) from the Household, Income, and Labour Dynamics in Australia (HILDA) Survey and analyzed sociodemographic and personality variables (Big Five, locus of control) as well as the extent of problem gambling. Findings showed that male sex and a lower level of education were related to problem gambling, but personality traits were predictive of problem gambling over and above sociodemographic variables. Specifically, a low level of emotional stability, an external locus of control, and, to a lesser extent, a low level of conscientiousness and a high level of extraversion were predictive of problem gambling, whereas openness and agreeableness were not. These results remained constant across various robustness analyses. Our findings reveal the importance of including personality traits when explaining gambling behavior.

## Introduction

Gambling can be defined as an activity that involves risking money on the result of something, such as a game or horse race, with the hope that one will make money [1]. It may be viewed along a severity continuum with recreational gambling activities at one end of the scale and *pathological gambling* (the term used in DSM-IV and ICD-10) [2, 3] or *gambling disorder* (the term used in DSM-5) [4] at the other end. The latter term refers to persistent and recurrent gambling behavior that leads to clinically significant distress and interference with functioning in major life domains [4]. In addition, the term *problem gambling* is often used to describe gambling that negatively affects functioning and relationships [5] without necessarily meeting DSM or ICD criteria for pathological gambling or gambling disorder.

Gambling is widespread: Statistics show that approximately 85% of U.S. adults have gambled at least once in their lives; 60% in the past year [6]. The worldwide lifetime prevalence of problem gambling is 2.3%, and the lifetime prevalence of pathological gambling is between 0.42 and 0.6% [7]. For Australia, there exist reliable statistics on gambling prevalence rates: In 2015, an estimated 39% of Australian adults gambled regularly. Among these regular gamblers, participation in lotteries was most common (76%), followed by instant scratch tickets (22%), and electronic gaming machines (21%) [8]. Whereas recreational gambling may help to improve physical and mental functioning, problem and pathological gambling have been found to be related to obesity, increased stress, and poorer physical health, violent behavior, and even suicidal thoughts or attempts [7, 9, 10].

What predicts individual differences in the extent of gambling? Research has shown that sociodemographic variables are related to gambling behavior. Across studies, male sex [11–15] and a lower educational level were predictive of problem gambling [11, 16]. With regard to age, findings have been more mixed. Studies have found higher age to be related to problem gambling when samples consisted of young adults [13] (but see [17] for similar findings in an older sample). By contrast, in samples involving a wider age span, age was not significantly related to problem gambling [16, 18]. In addition to sociodemographic variables, personality factors such as the Big Five and locus of control were found to be associated with gambling behavior.

### Prior research on personality and problem gambling

Researchers have examined the personality correlates of gambling using various methods, ranging from reviews [19] to empirical studies. The latter have been based on small samples [16, 20, 21], large data sets [13, 22], or even population-wide or birth-cohort studies [12, 23]. Studies have involved non-treatment-seeking participants, community samples [18, 24], and pathological gamblers, including patients with a DSM diagnosis [12, 16, 17, 20–22, 25].

Personality traits such as the Big Five have been examined trait by trait [17, 20] or simultaneously, using regression analyses [18, 24], in order to understand their relations to gambling participation and problem gambling. Despite the existence of numerous studies on the relation between personality and gambling, findings have been mixed as the following brief overview shows: Neuroticism or emotional instability was not associated [20] or was positively associated with problem gambling [14, 17, 18, 22, 24, 25]. Extraversion or positive affectivity was not associated [14, 17, 24, 25], was positively associated [13], or was negatively associated with problem gambling [20] (only for older participants: [18]). Openness to experience was not associated [14, 17, 24], or was negatively associated with problem gambling [20, 25] (especially for men: [18]). Similarly, agreeableness showed either no relation [17, 20] or a negative relation to problem gambling [14, 18, 22, 24, 25]. Across various studies, conscientiousness was found to be negatively related to problem gambling [14, 17, 20, 22, 24, 25] (for younger participants only: [18]). When the Big Five were examined simultaneously, high levels of neuroticism and low levels of openness manifested unique relations with problem gambling [16, 18]. In other research, specific facets of neuroticism, agreeableness, and conscientiousness were predictive of problem gambling [24].

Locus of control refers to the perception of the extent to which an individual can control events in his or her life [26]. So far, the findings on the relation between locus of control and problem gambling have been contradictory. Whereas some studies revealed an association between problem gambling and an external expectancy orientation—the belief that events in one’s life are controlled by factors beyond one’s influence or control [27, 28], other research did not find such a relation [29–31]. In sum, the pattern of results on the association between personality and gambling has been inconsistent.

### The present research

The objective of the present study was to investigate the association between personality and problem gambling using a large, representative sample. We predicted the extent of problem gambling from sociodemographic variables and personality traits (i.e., the Big Five and locus of control). All variables were investigated in conjunction because traits naturally co-occur. Sociodemographic variables were taken into account because personality traits are related to sex, age, and educational level. For example, neuroticism is positively related to female sex [32–34], extraversion is modestly negatively correlated with age [35, 36], and openness is sometimes positively correlated with education [37]. Furthermore, as was mentioned above, sociodemographic variables are also associated with problem gambling.

This reasoning leads to three main questions that guided our research: First, what sociodemographic variables and personality traits are associated with problem gambling? Second, what personality traits are uniquely predictive of problem gambling? And third, are the findings robust against variations in computational methods such as (a) logarithmizing the dependent variable problem gambling, (b) studying active gamblers only, and (c) investigating only cases with a level of problem gambling above zero?

## Methods

The data used in the present study were provided by the Household, Income, and Labour Dynamics in Australia (HILDA) Survey, a large, longitudinal, household-based panel study that examines economic and personal well-being, labor market dynamics, and family life. The HILDA survey has been conducted since 2001 [38], and the data are close to nationally representative when compared with population estimates [39]. The survey waves take place annually, and all data sets are matched via a unique identifier for each person. The present study used data collected in 2013 (Wave 13) and in 2015 (Wave 15). All research was performed in accordance with relevant guidelines/regulations. Informed consent was obtained from all participants and/or their legal guardians. The research was approved by Melbourne Institute. The data that support the findings of this study are available from the Melbourne Institute. Restrictions apply to the availability of these data, which were used under license for this study. Data are available at https://melbourneinstitute.unimelb.edu.au/hilda with the permission of the Melbourne Institute. The authors confirm that they did not have any special access privileges.

### Participants

The data set contained *N* = 23,292 valid cases (48% male, 52% female) with a mean age of 37 years (*SD* = 23.02; Range: 0 to 99). As monetized gambling is illegal under the age of 18, in our analyses, we included only individuals who were 18 or older (*N* = 17,688). Further, we included only individuals who provided valid answers to all analyzed variables. Thus, our sample consisted of *N* = 12,556 individuals (46% male, 54% female) with a mean age of 48 years (*SD* = 18, Range: 18 to 98).

### Measures

#### Sociodemographic variables

We analyzed age, sex (1 = *male*, 2 = *female*), and educational level as reported in 2015. Education was assessed using multiple categories that were based on the Australian Standard Classification of Education: 1 = undetermined, 2 = Year 11 and below, 3 = Year 12, 4 = Cert III or IV, 5 = Diploma, 6 = Bachelor or honors, 7 = Grad Diploma, grad certificate, 8 = Postgrad–masters or doctorate (for details: [40]).

#### Personality

The Big Five personality traits were assessed with a 36-item version of the Big Five Personality Inventory [41]. Because the factor structure could be improved by focusing on a subset of 28 items [42], we used this subset of items for our analyses. Each Big Five dimension was assessed using four to six items: *emotional stability* (envious, fretful, jealous, moody, temperamental, and touchy, all reverse coded; α = .80); *extraversion* (extraverted, lively, talkative; and reverse coded: bashful, quiet, and shy; α = .76); *openness to experience* (deep, complex, creative, imaginative, intellectual, and philosophical; α = .74), *agreeableness* (cooperative, kind, sympathetic, and warm; α = .78), and *conscientiousness* (efficient, orderly, systematic; and reverse coded: disorganized, inefficient, and sloppy; α = .79). Items were answered on 7-point Likert-type scales ranging from 1 (*does not describe me at all*) to 7 (*describes me very well*). Intercorrelations of the Big Five dimensions ranged from *r* = .07 (*p* < .001; extraversion and openness) to *r* = .32 (*p* < .001; emotional stability and conscientiousness). The data were collected in Wave 13.

Locus of control was assessed with seven items that were answered on 7-point Likert-type scales ranging from 1 (*strongly disagree*) to 7 (*strongly agree*) [43]. After reverse coding where necessary and aggregating the items, locus of control was used as a continuous variable (α = .84), with higher scores representing an external locus of control and lower scores representing an internal locus of control. Intercorrelations with the Big Five dimensions ranged from *r* = -.01 (*p* = .351) for openness to *r* = -.29 (*p* < .001) for emotional stability. The data were collected in Wave 15.

#### Gambling

To assess the extent of problem gambling, participants were presented nine items that captured problematic gambling behavior and the adverse consequences of gambling experienced in the past 12 months (Problem Gambling Severity Index [44]; e.g., *Have you felt guilty about the way you gamble or what happens when you gamble*?). These items were answered using a 4-point Likert-type scale ranging from 0 (*never*) to 3 (*almost always*). All nine items were summed to form the continuous variable problem gambling (*M* = 0.27, *SD* = 1.46, Range: 0 to 27, α = .92; see S1 File for a categorial analysis and a comparison with prevalence rates).

In order to analyze a subsample of active gamblers only, we used a measure of whether participants had spent money on gambling activities. To measure the extent to which money had been spent on different gambling activities, participants were presented 10 gambling activities (instant scratch tickets, bingo, lotto or lottery games, keno, private betting, poker, casino table games, poker machines or slot machines, betting on horse or dog races, betting on sports) and asked to indicate whether they tended to spend any money on these activities in a typical month (*no/yes*; *M* = 0.64, *SD* = 0.97, Range: 0 to 10). The data were collected in Wave 15. Descriptive statistics and intercorrelations for all measures are presented in S1 and S2 Tables in S1 File.

### Analyses

In a first step, we computed zero-order correlations of problem gambling with sociodemographic and personality variables. These statistics provide a first impression of the bivariate relations between our dependent and independent variables. In a second step, we computed partial correlations of problem gambling with personality variables, controlling for sociodemographic variables. Using this statistics, we were able to understand the extent to which problem gambling was linked to each personality variable over and above the influence of sociodemographic variables. In the following steps, all Big Five as well as locus of control were investigated in conjunction using regression analyses: We computed a regression, predicting problem gambling from age, sex, and education as well as the Big Five and locus of control, entered in two steps into the regression equation. By employing this procedure, we were able to determine the effect that each independent variable had on problem gambling over and above the other ones. For instance, we could identify the extent to which emotional stability was predictive of problem gambling when the influence of all other variables (e.g., sex, extraversion, and others) was held constant. Because problem gambling showed an excess of zeroes (extremely left-skewed distribution) and we could not separate non-gamblers from non-problem-gamblers, we computed three additional models to analyze whether our findings from the first regression analysis were robust: (a) We logarithmized the problem gambling variable in order to reduce the skewness of the distribution [45] and repeated the regression analysis as described above. (b) We created a subsample that included only participants who indicated that they had spent money on one or more of the given gambling activities. Using this subsample (*n* = 5,051, with a mean age of 52 years, *SD* = 16.89, Range: 18 to 97; 48% female), we again predicted problem gambling from sociodemographic variables and personality. By using this procedure, we analyzed only participants who had spent money on gambling activities (i.e., non-gamblers were excluded). (c) We repeated our regression analysis with participants who indicated any value above zero on the problem gambling scale (i.e., 1 to 27; *n* = 921, with a mean age of 47 years, *SD* = 17.62, Range: 18 to 96; 38% female). This procedure allowed us to examine whether a larger extent of problem gambling went along with certain sociodemographic variables and personality traits without being biased by an excess of zeroes on the problem gambling scale.

## Results

### What personality traits are associated with problem gambling?

To begin with sociodemographic variables, male sex and a lower level of education were correlated with problem gambling, whereas age showed no association with problem gambling (see Table 1). Focusing on personality correlates, high external locus of control, low emotional stability, low conscientiousness, and, to a lesser extent, low agreeableness were associated with problem gambling. These relations remained significant even when we controlled for all sociodemographic variables simultaneously. However, extraversion and openness to experience were not correlated with problem gambling.

**Table 1:** Associations of problem gambling with sociodemographic and personality variables.

	Problem gambling			
	*r*	*p*	*r*_part_	*p*
**Age**	-.01	.*693*		
**Sex**	**-.06**	*<* .*001*		
**Education**	**-.07**	*<* .*001*		
**Emotional stability**	**-.08**	*<* .*001*	**-.08**	*<* .*001*
**Extraversion**	-.01	.*208*	-.00	.*651*
**Openness to experience**	-.02	.*103*	-.00	.*952*
**Agreeableness**	**-.05**	*<* .*001*	**-.03**	.*005*
**Conscientiousness**	**-.07**	*<* .*001*	**-.06**	*<* .*001*
**External locus of control**	**.09**	*<* .*001*	**.09**	*<* .*001*

*N* = 12,556 for zero-order correlations (*r*) and partial correlations (*r*_part_; controlled for sociodemographic variables). Problem gambling was assessed with nine items and used as a continuous variable. Sex was coded as 1 = male and 2 = female. For education, a higher number reflects a higher level of education.

### What personality traits are uniquely predictive of problem gambling?

Initially, the sociodemographic variables low level of education and male sex were identified as predictors of problem gambling (see Table 2). However, the prediction could be improved by adding personality variables: In addition to sex and level of education, low emotional stability, high external locus of control, and, to a lesser extent, low conscientiousness and high extraversion were unique predictors of problem gambling over and above all other variables. Thus, especially with regard to extraversion and agreeableness, the pattern of results changed slightly when all predictors were added simultaneously into the regression analysis in comparison to the zero-order or partial correlations.

**Table 2:** Predicting problem gambling from sociodemographic and personality variables.

		Beta	*p*
**1**	**Constant**		*<* .*001*
	**Age**	-.01	.*187*
	**Sex**	**-.06**	*<* .*001*
	**Education**	**-.07**	*<* .*001*
**2**	**Constant**		*<* .*001*
	**Age**	.01	.*523*
	**Sex**	**-.07**	*<* .*001*
	**Education**	**-.06**	*<* .*001*
	**Emotional stability**	**-.07**	*<* .*001*
	**Extraversion**	**.03**	.*003*
	**Openness to experience**	-.02	.*114*
	**Agreeableness**	.00	.*841*
	**Conscientiousness**	**-.03**	.*003*
	**External locus of control**	**.06**	*<* .*001*

Regression analyses with *N* = 12,556. Problem gambling was assessed with nine items and used as a continuous variable. Sex was coded as 1 = male and 2 = female. For education, a higher number reflects a higher level of education. In Step 1, *R* = .10, *R*^*2*^ = .01; in Step 2, *R* = .15, *R*^*2*^ = .02.

### Are the findings robust against various evaluation methods?

As shown in Table 3, when the analyses were repeated using problem gambling as a logarithmized variable, the sociodemographic variables low level of education and male sex were identified as predictors of problem gambling. Again, the prediction could be improved by adding personality variables: In addition to sex and level of education, low emotional stability, high external locus of control, high extraversion, and, to a lesser extent, low conscientiousness and low openness were unique predictors of problem gambling over and above all other variables.

**Table 3:** Predicting problem gambling from sociodemographic and personality variables using additional analyses.

		Logarithmized		Spent money		Above zero	
		*N* = 12,556		*N* = 5,051		*N* = 921	
		Beta	*p*	Beta	*p*	Beta	*p*
**1**	**Constant**		*<* .*001*		*<* .*001*		*<* .*001*
	**Age**	-.00	.*743*	**-.07**	*<* .*001*	-.06	.*068*
	**Sex**	**-.08**	*<* .*001*	**-.07**	*<* .*001*	-.02	.*644*
	**Education**	**-.09**	*<* .*001*	**-.08**	*<* .*001*	**-.09**	.*005*
**2**	**Constant**		*<* .*001*		*<* .*001*		*<* .*001*
	**Age**	.02	.*082*	**-.05**	*<* .*001*	-.03	.*440*
	**Sex**	**-.09**	*<* .*001*	**-.08**	*<* .*001*	-.03	.*407*
	**Education**	**-.07**	*<* .*001*	**-.07**	*<* .*001*	**-.08**	.*015*
	**Emotional stability**	**-.07**	*<* .*001*	**-.07**	*<* .*001*	**-.13**	.*001*
	**Extraversion**	**.04**	*<* .*001*	**.04**	.*018*	.03	.*468*
	**Openness to experience**	**-.02**	.*018*	-.01	.*548*	-.00	.*951*
	**Agreeableness**	.00	.*769*	.02	.*234*	-.01	.*888*
	**Conscientiousness**	**-.03**	.*001*	**-.05**	.*002*	-.06	.*074*
	**External locus of control**	**.06**	*<* .*001*	**.09**	*<* .*001*	**.12**	.*001*
	**Step 1**	R = .12, R^2^ = .01		R = .12, R^2^ = .02		R = .11, R^2^ = .01	
	**Step 2**	R = .17, R^2^ = .03		R = .19, R^2^ = .04		R = .24, R^2^ = .06	

Logarithmized: The problem gambling variable was logarithmized in order to reduce the skewness of the distribution, and a regression analysis was performed (predicting problem gambling from sociodemographic variables and personality). Spent money: We created a subsample that included only participants who indicated that they had spent money on one or more of the given gambling activities (i.e., non-gamblers were excluded); using this subsample, a regression analysis was performed (predicting problem gambling from sociodemographic variables and personality). Above zero: We created a subsample that included only participants who indicated any value above zero on the problem gambling scale; using this subsample, a regression analysis was performed (predicting problem gambling from sociodemographic variables and personality). Sex was coded as 1 = male and 2 = female. For education, a higher number reflects a higher level of education.

When the analyses were performed using a subsample of participants who agreed that they had spent money on at least one game, the sociodemographic variables low level of education, male sex, and younger age were identified as predictors of problem gambling. Again, the prediction could be improved by adding personality variables. In addition to sex, education, and age, the extent of problem gambling was predicted by high external locus of control, low emotional stability, and, to a lesser extent, low conscientiousness and high extraversion, when all other predictors were held constant.

Finally, when the analyses were performed using a subsample of participants who indicated a level of problem gambling above zero, the sociodemographic variable low level of education was identified as a predictor of problem gambling. Again, the prediction could be improved by adding personality variables. In addition to education, the extent of problem gambling was predicted by low emotional stability, high external locus of control, and, to a lesser extent, low conscientiousness, when all other predictors were held constant.

## Discussion

The purpose of our research was to understand the relation between personality and problem gambling using a large data set. Our analyses allowed us not only to gain important insights into the relation of gambling behavior with sociodemographic and personality variables but also to specify the unique influence of personality on problem gambling over and above the influence of sex, age, and education. Our study revealed very consistent findings across various evaluation methods. Across all analyses, a low level of education was uniquely predictive of problem gambling. Likewise, male sex was consistently related to problem gambling (Due to a smaller sample size, findings were only marginally significant in the subsample of those participants who indicated a level of problem gambling above zero.). In all but one analysis, age was not associated with problem gambling.

Regarding personality variables, across analyses, a low level of emotional stability, a high level of external locus of control, and a low level of conscientiousness were robustly identified as unique predictors of problem gambling. High extraversion was weakly related to problem gambling (Due to a smaller sample size, findings were only marginally significant in the subsample of those participants who indicated a level of problem gambling above zero.). Agreeableness was not uniquely predictive of problem gambling. The findings for openness were similar to those for agreeableness. Across regression analyses, when personality variables were taken into account, the amount of variance that was explained in problem gambling was enhanced over and above what was explained by the sociodemographic variables alone. This finding underlines the importance of considering personality variables when trying to understand various gambling behaviors. In sum, our findings confirm and expand the results of prior studies.

However, our study goes far beyond a simple replication of prior research for several reasons: (a) We used a large representative sample of more than 12,500 individuals, including a wide range of age and educational levels as well as measures of different personality traits (Big Five, locus of control). (b) We assessed the extent of problem gambling with a commonly used instrument (Problem Gambling Severity Index, PGSI [44]) and used it as a continuous variable instead of forming artificial categories. Thus, we used the full set of available information and could interpret our results accordingly (e.g., the lower the level of emotional stability, the higher the extent of problem gambling). (c) Our analyses included not only zero-order correlations and partial correlations (controlling for sociodemographic variables) between personality traits and problem gambling but also regression analyses. The regressions allowed us to estimate the influence of each personality trait on the prediction of problem gambling, over and above the remaining variables. Thus, we were able to specify the comparatively most influential personality traits for the prediction of problem gambling, namely, emotional stability and external locus of control. (d) We checked the robustness of our findings by log-transforming our dependent variable and by analyzing subsamples that met strict criteria (spending money on gambling activities, showing a problem gambling level above zero). Thus, we minimized potential errors that stemmed from a specific method of analysis.

Despite these advantages, our study surely comes with limitations. Due to the nature of household-based panels, we had to rely on self-reports instead of objective measures of personality and gambling behavior. Future studies may implement ways of collecting observed data on personality (e.g., acquaintance reports) and gambling behavior (e.g., frequency of using a credit card to make gambling-related payments). In addition, the measure to access our dependent variable, the PGSI, is perceived rather critically by some research groups [46–48]. Precisely, there seems to be poor discriminant validity for low-risk and moderate-risk categories [46]. Further, as known for other self-report measures, recall biases and language difficulties may produce misclassifications to the predefined categories [48]. However, both arguments against the use of the PGSI do not fully apply to our analyses because we used the PGSI as a continuous variable rather than a categorial score. On the other hand, the PGSI comes with good psychometric properties such as an adequate temporal stability and is thus well-regarded by researchers and clinicians in the field [46, 48]. Due to the correlational nature of our study, we could not definitively separate causes from consequences. For instance, an individual low on emotional stability may engage in gambling behavior in order to distract him- or herself from daily hassles and to calm down. Likewise, an individual who gambles more and more often may experience a decline in his or her emotional stability as a result of financial and relationship problems. However, in our study, personality traits (except external locus of control) were measured prior to the assessment of problem gambling (see [49] for an exceptional example on how personality predicts betting behavior up to 34 years later). Thus, it seems plausible to assume that stable personality factors were the causes of behavior such as problem gambling. The national pathological gambling prevalence estimates in Western countries range from 0.5% in New Zealand [50] to 2.1% in Australia [51]. We used a nationally representative data set of Australians for our analyses, and our findings revealed small to medium-sized effects of personality on problem gambling. Thus, studies on personality and problem gambling conducted in other countries may discover smaller effects due to smaller base rates of gambling.

Build upon the present findings, there are several interesting avenues for future research: (1) In addition to looking at global personality traits, researchers may study the prediction of problem gambling from more specific personality variables (e.g., motivations [52]) and concrete gambling-related behaviors and attitudes [53]. When including further (maybe more specific) predictors of problem gambling, one may investigate whether there is unique (or overlapping) variance of the global personality traits as compared to more specific gambling-related variables. (2) Researchers may analyze subgroups of gamblers [54, 55] or different forms of gambling such as gambling on cards, sports, or bingo [14]. This may help generate further treatments and prevention initiatives for vulnerable subgroups. (3) Finally, the relationships between personality and problem gambling should be examined longitudinally with the aims to (a) identify protective factors against the development of problem or pathological gambling and (b) implement policies to improve public health [7].

## Supporting information

S1 File (DOCX)
