# Supplementary material for: Associations of the Big Five and locus of control with problem gambling in a large Australian sample
Source: PLoS One. 2021 Jun 14;16(6):e0253046. doi: 10.1371/journal.pone.0253046 (PMC8202919; doi:10.1371/journal.pone.0253046)
Supplement: S1 File — (DOCX) [file pone.0253046.s001.docx]

**S1 File**

**S1 Table. Descriptive statistics and intercorrelations for all independent measures.**

|  |  |  |  |  | **Correlations with** | | | | | | | |
| --- | --- | --- | --- | --- | --- | --- | --- | --- | --- | --- | --- | --- |
|  | **Min** | **Max** | ***M*** | ***SD*** | **Sex** | **Edu** | **ES** | **E** | **O** | **A** | **C** | **Control** |
| Age | 18 | 98 | 48.21 | 18.02 | -.01 | -.11*** | .25*** | -.08*** | -.09*** | .09*** | .16*** | .08*** |
| Sex | 1 | 2 | 1.54 | .50 |  | -.01 | .02* | .11*** | -.05*** | .25*** | .09*** | .02+ |
| Education | 1 | 8 | 4.22 | 1.81 |  |  | .00 | .02+ | .25*** | .05*** | .08*** | -.10*** |
| Emotional stability | 1 | 7 | 5.20 | 1.08 |  |  |  | .19*** | -.21*** | .17*** | .32*** | -.29*** |
| Extraversion | 1 | 7 | 4.42 | 1.09 |  |  |  |  | .07*** | .19*** | .13*** | -.22*** |
| Openness to experience | 1 | 7 | 4.25 | 1.05 |  |  |  |  |  | .26*** | .07*** | -.01 |
| Agreeableness | 1 | 7 | 5.46 | .90 |  |  |  |  |  |  | .29*** | -.15*** |
| Conscientiousness | 1 | 7 | 5.15 | 1.02 |  |  |  |  |  |  |  | -.24*** |
| External locus of control | 1 | 7 | 2.56 | 1.15 |  |  |  |  |  |  |  |  |

*Note. N* = 12,556. Sex was coded as 1 = male and 2 = female. For education, a higher number reflects a higher level of education.

+ *p* < .10. * *p* < .05. ** *p* < .01. *** *p* < .001.

**S2 Table. Descriptive statistics and intercorrelations for all dependent measures.**

|  |  |  |  |  |  | **Correlations with** | | |
| --- | --- | --- | --- | --- | --- | --- | --- | --- |
|  | **N** | **Min** | **Max** | ***M*** | ***SD*** | PG—logarithmized | PG—spent money | PG—above zero |
| Problem gambling | 12,556 | 0 | 27 | .27 | 1.46 | .93*** | 1.00*** | 1.00*** |
| Problem gambling—logarithmized | 12,556 | 0 | 3.33 | .10 | .38 |  | .93*** | .93*** |
| Problem gambling—spent money | 5,051 | 0 | 27 | .56 | 1.96 |  |  | 1.00*** |
| Problem gambling—above zero | 921 | 1 | 27 | 3.72 | 4.01 |  |  |  |

*Note.* Problem gambling was assessed with nine items and was used as a continuous variable. Logarithmized: The problem gambling variable was logarithmized in order to reduce the skewness of the distribution. Spent money: We created a subsample that included only participants who indicated that they had spent money on one or more of the given gambling activities (i.e., non-gamblers were excluded). Above zero: We created a subsample that included only participants who indicated any value above zero on the problem gambling scale.

**S3 Table. Distribution of the Problem Gambling Severity Index categories across samples.**

|  |  | **Full sample** | | **Spent money** | |
| --- | --- | --- | --- | --- | --- |
|  |  | *N* = 12,556 | | *N* = 5,051 | |
| PGSI categories |  | N | % | N | % |
| 0 |  | 11,635 | 92.7 | 4,253 | 84.2 |
| 1 |  | 496 | 4.0 | 426 | 8.4 |
| 2 |  | 309 | 2.5 | 285 | 5.6 |
| 3 |  | 116 | .9 | 87 | 1.7 |

*Note.* Problem gambling was assessed with nine items and was used as a *categorial* variable. The Problem Gambling Severity Index (PGSI; [1]) categories are defined as follows: 0 = non-problem gamblers (0 points), 1 = low-risk gamblers (1-2 points), 2 = moderate-risk gamblers (3-7 points), 3 = problem gamblers (8 and over points). Subsample spent money: We created a subsample that included only participants who indicated that they had spent money on one or more of the given gambling activities (i.e., non-gamblers were excluded). These rates are comparable to general prevalence rates of gambling in Australia: 3.0 per cent low-risk gambling (95% CI = 2.2 - 4.0), 1.9 per cent moderate-risk gambling (95% CI = 1.3 - 2.6), and 0.4 per cent problem gambling (95% CI = 0.2 - 0.8; *N* = 2,000; [2]).

**References**

1. Ferris JA, Wynne HJ. The Canadian problem gambling index. Ottawa, ON: Canadian Centre on Substance Abuse 2001.

2. Dowling NA, Youssef GJ, Jackson AC, Pennay DW, Francis KL, Pennay A, et al. National estimates of Australian gambling prevalence: Findings from a dual‐frame omnibus survey. Addiction 2016; 111:420-435. doi:/10.1111/add.13176
